# Quercetin Attenuates Cardiac Hypertrophy by Inhibiting Mitochondrial Dysfunction Through SIRT3/PARP-1 Pathway

**DOI:** 10.3389/fphar.2021.739615

**Published:** 2021-10-28

**Authors:** Wen-Jing Chen, Yan Cheng, Wen Li, Xiao-Kang Dong, Jian-liang Wei, Chuan-Hua Yang, Yue-Hua Jiang

**Affiliations:** ^1^ First Clinical Medical College, Shandong University of Traditional Chinese Medicine, Jinan, China; ^2^ Cardiovascular Department, Affiliated Hospital of Shandong University of Traditional Chinese Medicine, Jinan, China; ^3^ College of Traditional Chinese Medicine, Shandong University of Traditional Chinese Medicine, Jinan, China; ^4^ Central Laboratory, Affiliated Hospital of Shandong University of Traditional Chinese Medicine, Jinan, China

**Keywords:** quercetin, mitochondrial function, Sirtuin3, poly (ADP-ribose) polymerase-1, cardiac hypertrophy

## Abstract

Cardiac hypertrophy is an important characteristic in the development of hypertensive heart disease. Mitochondrial dysfunction plays an important role in the pathology of cardiac hypertrophy. Recent studies have shown that sirtuin 3 (SIRT3)/poly (ADP-ribose) polymerase-1 (PARP-1) pathway modulation inhibits cardiac hypertrophy. Quercetin, a natural flavonol agent, has been reported to attenuate cardiac hypertrophy. However, the molecular mechanism is not completely elucidated. In this study, we aimed to explore the mechanism underlying the protective effect of quercetin on cardiac hypertrophy. Spontaneously hypertensive rats (SHRs) were treated with quercetin (20 mg/kg/d) for 8 weeks to evaluate the effects of quercetin on blood pressure and cardiac hypertrophy. Additionally, the mitochondrial protective effect of quercetin was assessed in H9c2 cells treated with Ang II. SHRs displayed aggravated cardiac hypertrophy and fibrosis, which were attenuated by quercetin treatment. Quercetin also improved cardiac function, reduced mitochondrial superoxide and protected mitochondrial structure *in vivo*. *In vitro*, Ang II increased the mRNA level of hypertrophic markers including atrial natriuretic factor (ANF) and β-myosin heavy chain (β-MHC), whereas quercetin ameliorated this hypertrophic response. Moreover, quercetin prevented mitochondrial function against Ang II induction. Importantly, mitochondrial protection and PARP-1 inhibition by quercetin were partly abolished after SIRT3 knockdown. Our results suggested that quercetin protected mitochondrial function by modulating SIRT3/PARP-1 pathway, contributing to the inhibition of cardiac hypertrophy.

## Introduction

Cardiac hypertrophy is a crucial characteristic of hypertensive heart disease that mainly involves cardiomyocyte hypertrophy in the early stage. Even though this may be a compensatory response initially to maintain function and efficiency in response to pressure overload, persistent increase in hypertrophy ultimately leads to ventricular dilatation and heart failure (Nakamura and Sadoshima, 2018). Therefore, ameliorating cardiac hypertrophy is the emphasis of clinical hypertension treatment.

Mitochondrial function, including reactive oxygen species (ROS) generation and detoxification, energy metabolism, mitochondrial biogenesis, mitophagy and dynamics, plays a vital role in maintaining cellular homeostasis of cardiomyocytes (Nunnari and Suomalainen, 2012). In recent years, mitochondrial dysfunction has emerged as one of the main pathogenic mechanisms underlying the development of cardiac hypertrophy (McDermott-Roe et al., 2011; Facundo et al., 2017). Reduced energy production and increased oxidative stress, as the main consequences of mitochondrial dysfunction, result in cardiomyocyte death and fibrosis, leading to progress of maladaptive hypertrophy (Forte et al., 2019). These findings suggest that modulating mitochondrial function may be considered a valid therapeutic strategy in cardiac hypertrophy (Manolis et al., 2021).

Sirtuins (SIRTs) are a family of nicotinamide adenine dinucleotide (NAD)^+^–dependent deacetylases that consists of seven members (SIRT1—SIRT7) closely related to glucose/lipid metabolism, cell survival, energy metabolism, and NDA repair (Park et al., 2013). Among them, sirtuin 3 (SIRT3), localized in the mitochondrial matrix, regulates biological function of mitochondria as a vital stress-responsive protein deacetylase. In response to oxidative stress, SIRT3 is stimulated to deacetylate and activate superoxide dismutase (SOD2), leading to mitochondrial ROS clearance (Chen et al., 2011). The role of SIRT3 in cardiac hypertrophy has been widely examined both *in vivo* and *in vitro*. Recent studies demonstrate that SIRT3 knockout mice spontaneously develop myocardial fibrosis (Palomer et al., 2020), while SIRT3 overexpression partially attenuates the cardiac hypertrophic response by regulating the expression of antioxidant genes (Xiong et al., 2019) and mitochondrial function (Xu M. et al., 2019).

Poly (ADP-ribose) polymerases (PARPs) is a enzyme family composed of 17 members that can be activated by DNA damage. Poly (ADP-ribose) polymerase-1 (PARP-1), as the most abundant and ubiquitous member of the family, accounts for most of the PARP activity (Ryu et al., 2015). PARP-1 plays a crucial role in the physiological cellular functions such as DNA repair, transcription, bioenergetics, mtDNA maintenance, cell death and mitophagy (Kadam et al., 2020), using NAD^+^ as a substrate to transfer ADP-ribose units from NAD^+^ to form ADP-ribose polymers (PAR) (Hassa and Hottiger, 2008). Several studies have suggested that PARP-1 is closely associated with the development of cardiac hypertrophy. PARP-1 activity is significantly increased in diabetic cardiomyopathy (Waldman et al., 2018). In addition, treatment with the PARP-1 inhibitor AG-690/11026014 (6014) effectively prevents cardiomyocyte hypertrophy induced by Ang II (Feng et al., 2017). Importantly, a recent study reveals that SIRT3 interacts directly with PARP-1 and exerts protective effects against cardiomyocyte hypertrophy by deacetylating PARP-1 and inhibiting PARP-1 activity (Feng et al., 2020). Therefore, SIRT3/PARP-1 pathway regulation may represent a promising tool for improving mitochondrial function and treating cardiac hypertrophy.

Quercetin is a natural flavonol drug with many biological and health-promoting effects, including anti-inflammatory (Karuppagounder et al., 2016), antioxidant (Xu D. et al., 2019), antiatherosclerotic (Hung et al., 2015), antihypertensive (Elbarbry et al., 2020), anticancer (Reyes-Farias and Carrasco-Pozo, 2019), and anti-Alzheimer (Ebrahimpour et al., 2020) properties. In recent years, several studies have reported that quercetin protects against cardiac hypertrophy both *in vivo* and *in vitro* (Goncharov et al., 2016; Chen et al., 2018; Wang et al., 2020). Our previous study found that quercetin not only increases SIRT1 expression in aorta of ApoE^-/-^ mice but also decreases cellular apoptosis and ROS generation in ox-LDL-induced cellular senescence (Jiang et al., 2020). However, the molecular mechanism by which quercetin attenuates cardiac hypertrophy remains unclear.

The purpose of this study was to confirm that quercetin alleviated cardiac hypertrophy, focusing on the SIRT3/PARP-1 pathway both in spontaneously hypertensive rats (SHRs) and H9c2 cells.

## Materials and Methods

### Animals

Fifteen-week-old male SHRs (n = 16, weighing 280–320 g) and age/sex-matched Wistar-Kyoto (WKY) rats (n = 8, weighing 290–330 g) were obtained from Vital River Laboratory Animal Technology (Certificate: SCXK (Jing) 2016-0006, Beijing, China). The zoological study was performed in the Animal Experimental Center of the Affiliated Hospital of Shandong University of Traditional Chinese Medicine (Shandong, China) at controlled temperature (21 ± 1°C). The study was approved by the Animal Ethics Committee of the Affiliated Hospital of Shandong University of Traditional Chinese Medicine (Jinan, China), and the ethics approval number is AWE-2019-021.

After acclimatization for 1 week, 16 SHRs were randomly divided into the SHR (SHR, n = 8) and quercetin (Que, n = 8) groups; eight WKY rats were used as the normal control group (WKY, n = 8). According to a research and our previous study, intragastrical administration of quercetin at a dose of 20 mg/kg/d for 8–12 weeks prevented cardiac hypertrophy in established cardiac hypertrophic rat model and alleviated atherosclerotic lesion in ApoE^-/-^ mice, respectively (Chen et al., 2018; Jiang et al., 2020). Therefore, in this study, Rats in the quercetin group were administered 20 mg/kg/d quercetin (Meilun Biotechnology, Dalian, China) intragastrically, while the other two groups received the same volume of saline intragastrically for 8 weeks.

### Blood Pressure Measurement

Systolic blood pressure (SBP) and diastolic blood pressure (DBP) were measured by the tail-cuff method with Mouse and Rat Tail Cuff Blood Pressure Systems (MRBP-10, IITC Life Science, United States) before and after treatment every 2 weeks. Rats were placed on a heated platform (35°C) for 10 min to keep them warm before measurement. The measurements were repeated three times for each rat at each time point, with the mean value recorded.

### Echocardiography

Eight weeks after treatment, rats were anesthetized and their chests were shaved with a depilatory agent. Transthoracic echocardiography was performed using a Veterinary Ultrasonic system Ultrasound Scanner (M5 Vet, Mindray, Guangdong, China). Left ventricular structure and function were assessed by measuring left ventricular end-diastolic internal diameter (LVIDd), left ventricular end-diastolic posterior wall thickness (LVPWd), end-diastolic interventricular septal thickness (IVSd), left ventricular end-systolic internal diameter (LVIDs), left ventricular ejection fraction (LVEF) and left ventricular fractional shortening (LVFS) *via* two-dimensional and M-mode echocardiography.

### Histopathological Examination

After echocardiography, the heart was quickly extracted. The heart and body weights of each rat were recorded. Heart tissues were collected, fixed with 4% formaldehyde for 48 h at room temperature, embedded in paraffin, and sectioned at 5 μm. Sections were stained with hematoxylin and eosin (H&E) and Masson’s trichrome using standard protocols to assess general histology and interstitial fibrosis. In order to determine the cardiomyocyte cross-sectional area, heart sections were deparaffinized, rehydrated, and processed for antigen retrieval with EDTA antigen retrieval solution (Servicebio, G1206, Wuhan, China). Afer washes, sections were incubated with diluted wheat germ agglutinin (WGA) solution (Sigma-Aldrich, L4895, United States) at 37°C in the dark for 30 min, and then incubated with DAPI (Servicebio, G1012, Wuhan, China) at room temperature for 10 min. Following spontaneous fluorescence quenching, slides were mounted with anti-fade mounting medium. Images were captured using fluorescent microscope (Nikon, ECLIPSE C1, Japan). Four images were taken from four different rats in each group. The quantification of collagen volume fraction and cardiomyocyte cross-sectional area were analysed using Image-Pro Plus 6.0 software (Media Cybernetics, United States).

### Transmission Electron Microscopy

The mitochondrial ultrastructure was observed by TEM. Fresh cardiac tissues were cut into 1–3 mm blocks, fixed with 2% glutaraldehyde at 4°C overnight. After dehydration, resin penetration and embedding, the ultrathin sections were stained with 2% uranium acetate-saturated alcohol solution. Ultrastructural images were captured with a TEM (JEOL-1200, Japan).

### Mitochondrial Superoxide Measurement

Mitochondrial superoxide production was measured by MitoSOX™ Red mitochondrial superoxide indicator (Thermo Fisher (China), M36008, Shanghai, China) according to the manufacturer’s instructions. Freshly prepared frozen heart sections were incubated with 5 μM MitoSOX™ reagent working solution for 10 min at 37°C protected from light. Images were captured using fluorescence microscope (Nikon, Japan) with excitation and emission at 514 and 585 nm, respectively. Four images were taken from four different rats in each group. ImageJ (National Institutes of Health, Bethesda, Maryland, United States) was employed to analyze the relative levels of mitochondrial superoxide in the heart.

### Total Superoxide Dismutase, Malondialdehyde and Glutathione Peroxidase Assays

Blood samples were obtained from the inferior vena cava, and serum was obtained by centrifugation. Serum T-SOD, MDA and GSH-PX amounts were detected with the T-SOD assay (Jiancheng Biological Engineering Institute, A001-1, Nanjing, China), MDA assay (Jiancheng Biological Engineering Institute, A003-1) and GSH-PX assay (Jiancheng Biological Engineering Institute, A005-1) kits, respectively, according to the manufacturer’s instructions.

### Cell Culture

Immortalized rat myoblast H9c2 cells were purchased from Procell Life Science and Technology (Wuhan, China). H9c2 cells were cultured in DMEM (Procell) supplemented with 10% fetal bovine serum (Procell) and 1% penicillin/streptomycin (Procell) at 37°C in a humidified environment with 5% CO_2_. H9c2 cells cultured in DMEM were treated with 0.5, 1, and 2 μM quercetin, respectively. One hour after quercetin treatment, cells were treated with the hypertrophic agonist angiotensin II (Ang II; Solarbio, Beijing, China) at 0.1 μM. All cells were incubated for an additional 24 h before analysis.

### MTT Assay

Viability of H9c2 was determined by MTT assay. 20 μl MTT (5 mg/ml) was added to cultured cells in 96-well plate to incubated at 37°C for 4 h. Medium was replaced with 150 µl DMSO to dissolve the formazan crystals. Subsequently, the absorbance was measured using microplate reader (Thermo Scientific, Multiskan GO, United States) with wave length at 562 nm.

### Cell Transfection

A small interfering RNA targeting SIRT3 (siRNA-SIRT3) and siRNA-NC were designed and synthesized by GenePharma (Shanghai, China). H9c2 cells were cultured in six-well plates (3×10^5^ cells/well) with Opti-MEM (Thermo Fisher [China], Shanghai, China) overnight before transfection to ensure 80% cell confluence. Diluted Lipofectamine 3,000 (Thermo Fisher [China], Shanghai, China) and diluted siRNA were mixed, and incubated at room temperature for 20 min before adding to the corresponding plates. H9c2 cells were cultured at 37°C for 48 h, followed by western blot detection of transfection efficiency.

### Detection of Mitochondrial Membrane Potential (ΔΨm)

Mitochondrial membrane potential was detected using a fluorescence tetraethylbenzimidazolylcarbocyanine iodide (JC-1) assay kit (Beyotime, C2006, Shanghai, China). Collected H9c2 cells were incubated with JC-1 staining mixture at 37°C for 20 min and washed with JC-1 staining buffer twice. ΔΨm was analyzed on a flow cytometer (BD Accuri C6 Plus, United States) with excitation and emission at 485 and 590 nm, respectively. The aggregate/monomer (red/green fluorescence) ratio was used to measure mitochondrial membrane depolarization.

### Determination of Intracellular ATP Levels

Intracellular ATP levels were determined with an intracellular ATP assay kit (Beyotime, S0026, Shanghai, China) following the manufacturer’s instructions. H9c2 cells were lysed and centrifuged at 4°C for 10 min, and the resulting supernatant was used for further analysis. ATP detection reagent was added to each sample, and luminescence (RLU) was subsequently measured on a microplate reader (Bio-TEK, Synergy HTX, Vermont, United States). Finally, ATP levels were calculated according to a standard curve.

### Quantitative Real-Time PCR (q-PCR)

Total RNA was extracted from cultured H9c2 cells with TRIzol reagent (Invitrogen, Carlsbad, CA, United States) following the manufacturer’s protocol. Then, cDNA was synthesized and amplified by PCR with a Transcriptor Reverse Transcriptase kit (Roche, Basel, Switzerland). The mRNA levels of target genes were measured with ChamQ SYBR qPCR Master Mix (Vazyme, Nanjing, China) on the LightCyler 480 system (Roche, Basel, Switzerland). Glyceraldehyde 3-phosphate dehydrogenase (GAPDH) expression was used for normalization. The primers are listed in Supplementary Table S1.

### Western Blot

The concentration of total protein extracted from heart tissues and H9c2 cells were assessed with the BCA Protein assay kit (Cwbio, Jiangsu, China) and adjusted to 20 µg/µl. Then, 30 µg total protein per samples was separated by 12% sodium dodecyl sulfate polyacrylamide gel electrophoresis (SDS-PAGE) and subsequently transferred onto PVDF membranes. After blocking, the PVDF membranes were incubated with primary antibodies (Anti-Collagen I Rabbit pAb, GB11022, 1:1,000, Servicebio, Wuhan, China; Anti-alpha smooth muscle Actin Rabbit pAb, GB11044, 1:1,000, Servicebio, Wuhan, China; Anti-SIRT3 Rabbit pAb, GB11354, 1:1,000, Servicebio, Wuhan, China; PARP1 Rabbit pAb, A0942, 1:1,000, Abclonal, Wuhan, China; Anti-PAR mouse pAb, ab14459, 1:1,000, Abcam, Cambridge, United Kingdom) at 4°C overnight. After a washing step, the blots were incubated with horseradish peroxidase-conjugated goat anti-rabbit IgG or goat anti-mouse IgG (1:5,000) at room temperature for 2 h. Finally, immunoreactive bands were quantified by ImageJ (National Institutes of Health, Bethesda, Maryland, United States). GAPDH expression was used for normalization. Western blot was performed in three biological replicates.

### NAD^+^ Measurement

The level of NAD^+^ in H9c2 cells was determined using NAD^+^/NADH assay kit (Beyotime, S0175, Shanghai, China) with WST-8 according to the manufacturer’s instructions. In brief, 1 × 10^6^ cells were collected and lysed with 200 ul cold lysis buffffer to obtain sample. Afer reagent preparation, alcohol dehydrogenase working solution was added to 20 ul sample, and the suspension was incubated at 37°C for 10 min to obtain total NAD and NADH, respectively. Based on the relationship between NADH and the absorbance value measured at 450 nm, a standard curve was generated. Subsequently, the total NAD and NADH concentration were estimated according to the standard curve, and NAD^+^ concentration was calculated by subtracting NADH from total NAD.

### Statistical Analysis

Statistical analysis was performed using SPSS 26.0. Data are mean ± standard deviation (SD). Statistical differences among groups were determined by one-way analysis of variance (ANOVA) followed by least significant difference-*t* test (LSD-*t*). Differences were considered statistically significant at *p* < 0.05.

## Results

### Quercetin Decreases Blood Pressure in SHRs

**FIGURE 1:**
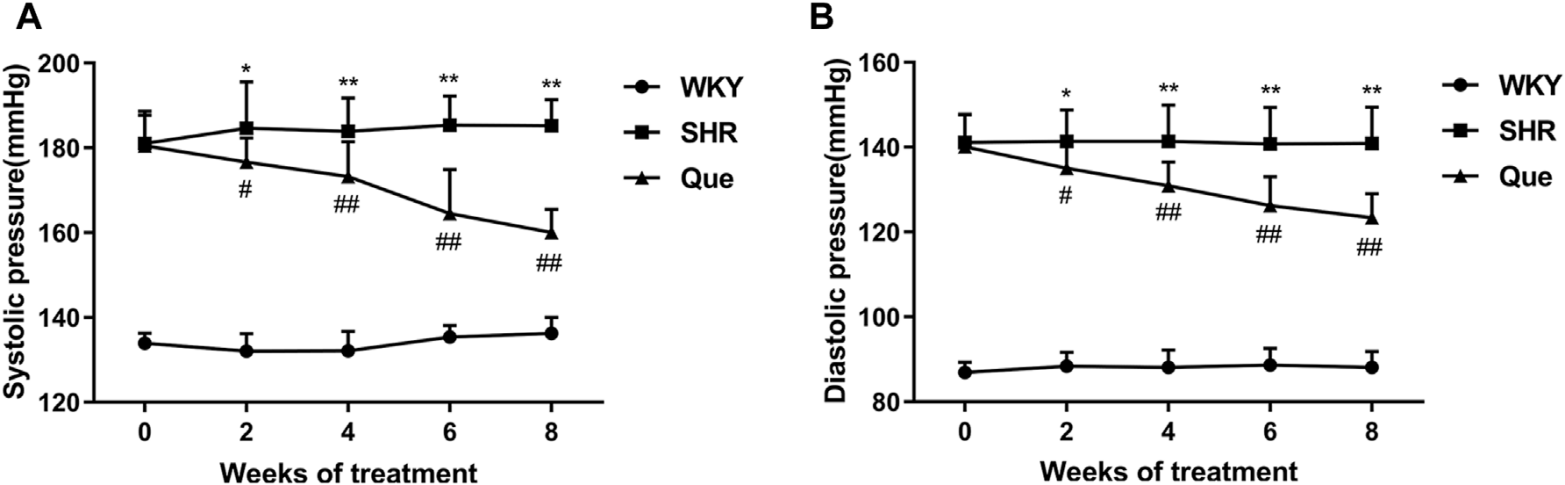
Quercetin decreases systolic blood pressure (SBP) and diastolic blood pressure (DBP) in spontaneously hypertensive rats (SHRs). **(A)** SBP in different rat groups at each time point (n = 8). **(B)** DBP in different rat groups at each time point (n = 8). Data are mean ± SD. ^*^ *p* < 0.05, ^**^ *p* < 0.01 *vs* WKY group; ^#^ *p* < 0.05, ^##^ *p* < 0.01 *vs* SHR group.

As shown in Figures 1A,B, SHRs had significantly higher SBP and DBP at each time point compared with WKY rats, and SBP and DBP levels were homogenous among SHRs before treatment. As expected, from the second week of treatment, both SBP and DBP in SHRs were significantly decreased by quercetin, suggesting an anti-hypertensive effect for quercetin.

### Quercetin Attenuates Cardiac Hypertrophy in SHRs

**FIGURE 2:**
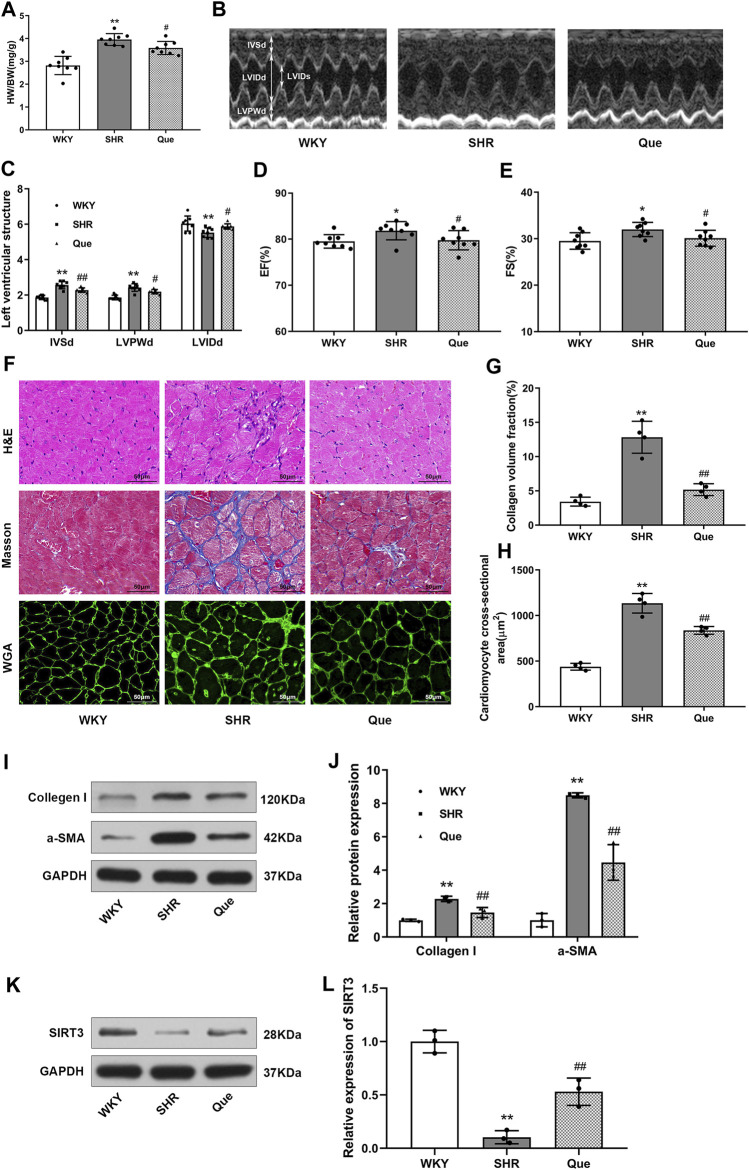
Quercetin attenuates cardiac hypertrophy in SHRs. **(A)** Heart weight to body weight ratios (HW/BW, n = 8). **(B)** Representative M-mode echocardiograms in WKY rats and SHRs. **(C–E)** Quantitative analysis of end-diastolic interventricular septal thickness (IVSd), left ventricular end-diastolic posterior wall thickness (LVPWd), left ventricular end-diastolic internal diameter (LVIDd), left ventricular ejection fraction (LVEF) and left ventricular fractional shortening (LVFS) (n = 8). **(F)** Representative histological images of the myocardium. In detail, hematoxylin and eosin (H&E) staining, Masson’s trichrome staining, and wheat germ agglutinin (WGA) staining in hearts from WKY rats and SHRs. Scale bar = 50 μm. (**G)** Quantitative analysis of collagen volume fraction (n = 4). **(H)** Quantitative analysis of cardiomyocyte cross-sectional area (n = 4). **(I,J)** Representative blots of collagen I and α-smooth muscle actin (α-SMA), and densitometric quantification after normalization to GAPDH levels (n = 3). Data are mean ± SD. ^*^ *p* < 0.05, ^**^ *p* < 0.01 *vs* WKY group; ^#^ *p* < 0.05, ^##^ *p* < 0.01 *vs* SHR group.

Next, we evaluated the efficacy of quercetin to attenuate cardiac hypertrophy in SHRs. As shown in Figure 2A, heart weight to body weight ratio (HW/BW) was higher in SHRs than WKY rats, which was related to increased LVPWd and IVSd, and decreased LVIDd in SHRs as revealed by the M-mode echocardiography. Whereas these pathological phenotype were significantly prevented in quercetin-treated rats (Figures 2B,C). In addition, SHRs developed increased LVEF and LVFS, which were also reversed to normal levels by quercetin administration (Figures 2D,E). Moreover, histological analysis showed elevated collagen volume fraction and cardiomyocyte cross-sectional area in SHRs compared with WKY rats, accompanied by increased collagen I and α-smooth muscle actin (α-SMA). However, quercetin treatment rescued these changes (Figures 2F–J). Since previous studies suggested a protective role of SIRT3 in cardiac hypertrophy (Pillai et al., 2015), we assessed SIRT3 levels in rats by western blot. SIRT3 levels were markedly reduced in SHRs, but significantly increased by quercetin treatment (Figures 2K,L). These results demonstrated that quercetin might attenuate cardiac hypertrophy by activating SIRT3-mediated signaling pathway *in vivo*.

### Quercetin Improves Mitochondrial Structure and Inhibits Oxidative Stress in SHRs

**FIGURE 3:**
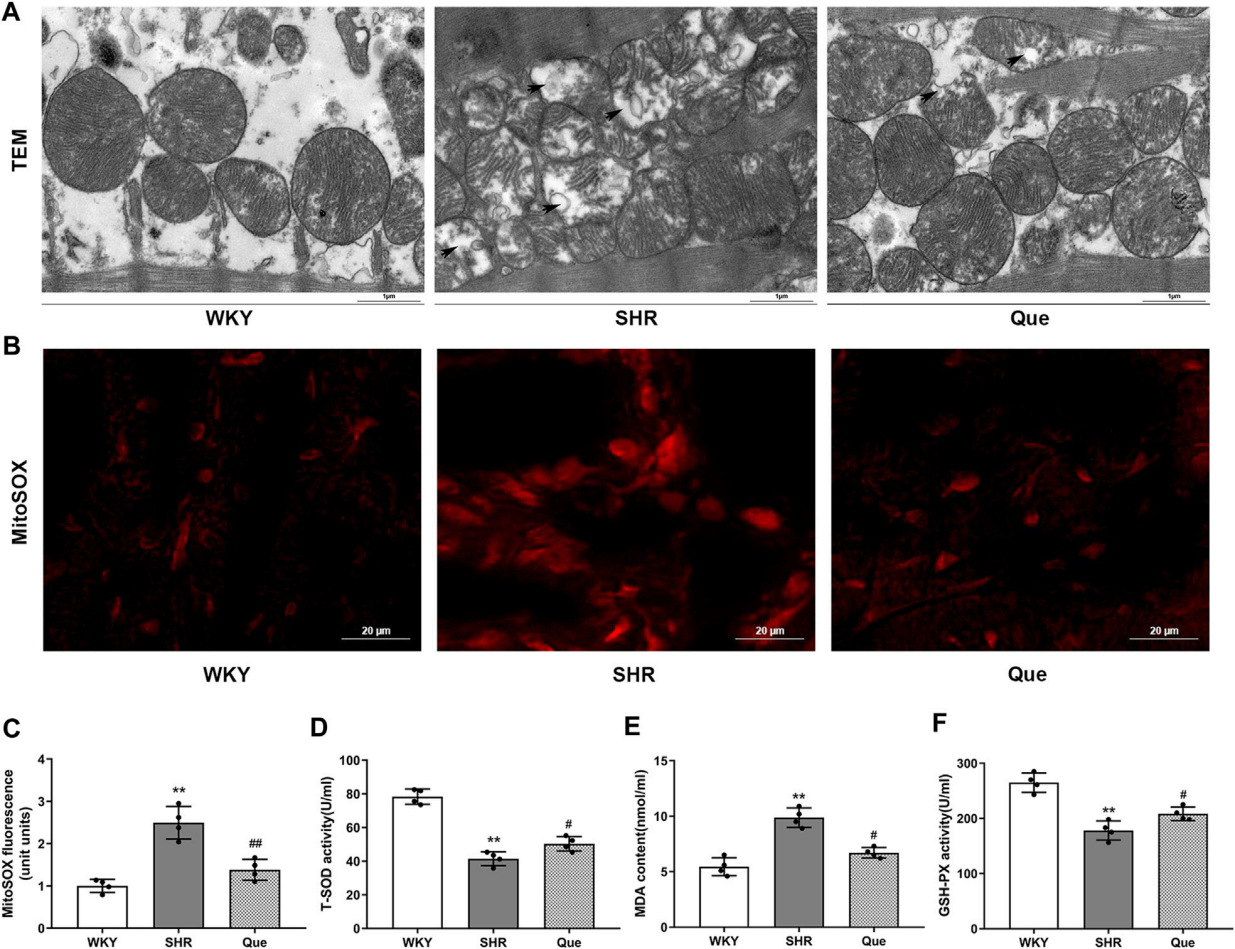
Quercetin improves mitochondrial structure and inhibits oxidative stress in SHRs. (**A)** Representative transmission electron microscopy (TEM) images of mitochondria in hearts from WKY rats and SHRs. Scale bar = 1 μm. **(B)** Representative microphotographs of MitoSOX staining in hearts from WKY rats and SHRs. Scale bar = 20 μm. **(C)** Quantitative analysis of the relative levels of mitochondrial superoxide in hearts from WKY rats and SHRs (n = 4). **(D–F)** Quantification of Total Superoxide dismutase (T-SOD), Malondialdehyde (MDA) and Glutathione peroxidase (GSH-PX) levels (n = 4). Data are mean ± SD. ^*^ *p* < 0.05, ^**^ *p* < 0.01 *vs* WKY group; ^#^ *p* < 0.05, ^##^ *p* < 0.01 *vs* SHR group.

Previous data demonstrated a close link between mitochondrial dysfunction and pressure overload-induced cardiac hypertrophy (Kumar et al., 2019). In order to assess the effect of quercetin on mitochondrial integrity and function in SHRs, we observed mitochondrial ultrastructure using TEM. Mitochondria in the hearts of WKY rats had regular morphology, with abundant cristae. Meanwhile, SHRs hearts showed defective mitochondrial organization, as indicated by swollen mitochondria with distorted cristae, even accompanied by mitochondrial membrane damage. However, quercetin treatment rescued these ultrastructure changes, with a relatively improved mitochondrial phenotype (Figure 3A). Based on the mitochondrial function in ROS generation, we detected mitochondrial superoxide with MitoSOX™ Red staining. As shown in Figure 3B, superoxide generation was significant increased in SHRs, which was decreased by quercetin treatment. In agreement, oxidative stress was exacerbated as determined by increased MDA and decreased T-SOD and GSH-PX in SHRs, whereas quercetin treatment significantly ameliorated oxidative stress (Figure 3C). These data confirmed that quercetin alleviated mitochondrial damage and oxidative stress *in vivo*.

### Quercetin Inhibits Ang II-Induced Hypertrophic Response in H9c2 Cells and Protects Mitochondrial Function *in Vitro*

**FIGURE 4:**
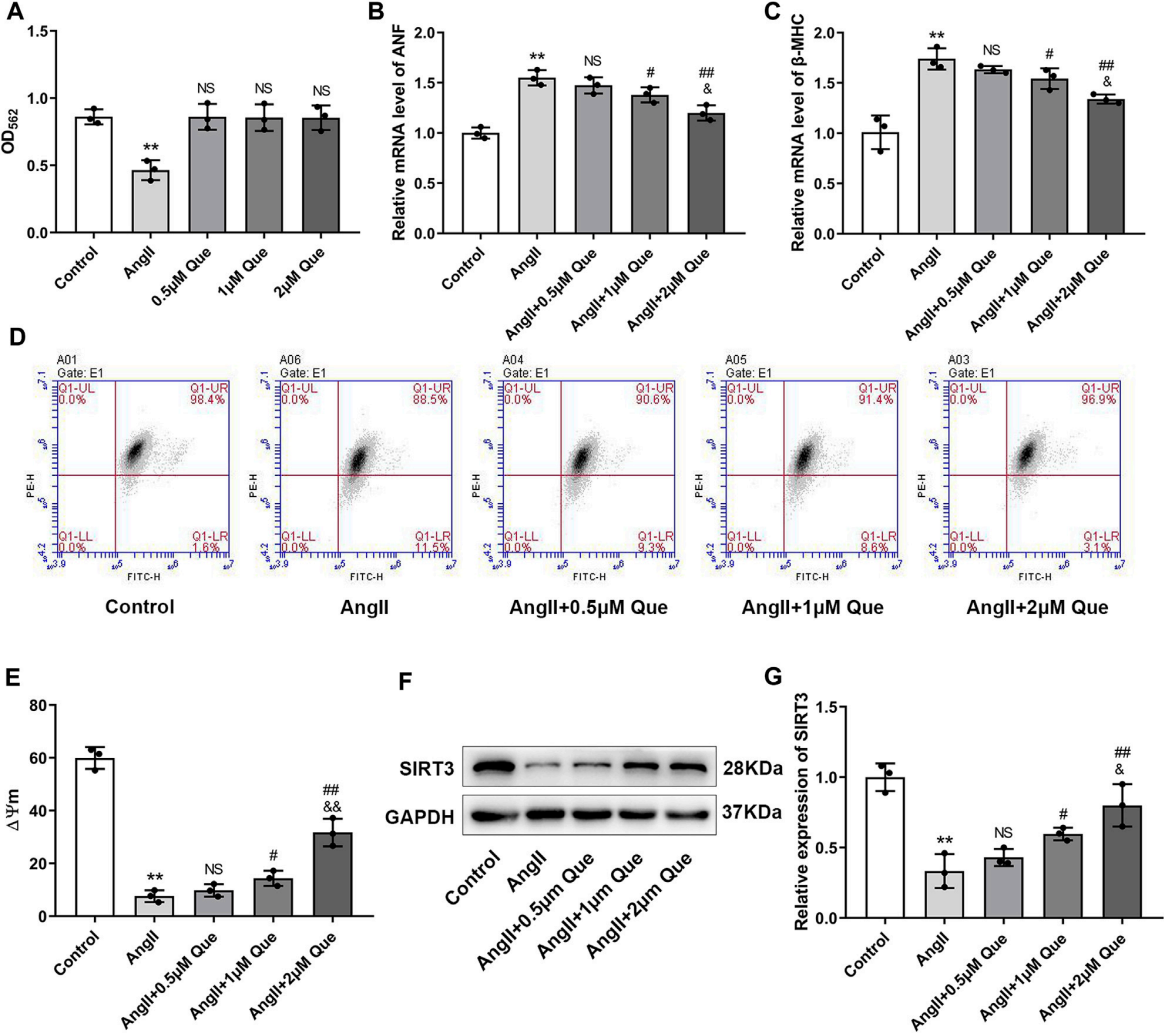
Quercetin inhibits Ang II-induced hypertrophic response in H9c2 cells and protects mitochondrial function *in vitro*. **(A)** Quantitative analysis of viability in H9c2 cells (n = 3). **(B,C)** Atrial natriuretic factor (ANF) and β-myosin heavy chain (β-MHC) mRNA levels determined by q-PCR (n = 3). **(D)** Mitochondrial membrane potential (ΔΨm) measurement with the JC-1 probe. **(E)** Quantification of ΔΨm by the aggregate/monomer ratio (n = 3). **(F,G)** Representative blots of the SIRT3 protein and densitometric quantification after normalization to GAPDH levels (n = 3). Data are mean ± SD. ^*^ *p* < 0.05, ^**^ *p* < 0.01 *vs* control group; ^#^ *p* < 0.05, ^##^ *p* < 0.01 *vs* Ang II group; ^NS^ *P* > 0.05 *vs* control/Ang II group; ^&^ *p* < 0.05, ^&&^ *p* < 0.01 *vs* Ang II + 1 μm Que group.

Before evaluating anti-hypertrophic effects of quercetin *in vivo*, we tested the cytotoxicity of quercetin by MTT assay. The MTT results showed that Ang II reduced viability of H9c2 cells by 46% compared with the control, while no significant reduction in cell viability was observed after quercetin intervention, indicating that there were no cytotoxic effects (Figure 4A). Subsequently, we investigated the hypertrophic response in Ang II-induced H9c2 cells by using hypertrophic markers, including atrial natriuretic factor (ANF) and β-myosin heavy chain (β-MHC), as indicators of cardiomyocyte hypertrophy. As shown in Figures 4B,C, Ang II-induced H9c2 cells showed increased mRNA levels of ANF and β-MHC compared with control cells, which were markedly reduced after quercetin treatment in a dose dependent manner.

To get further support for the protective effect of quercetin on mitochondrial function, we measured ΔΨm using JC-1. As shown in Figure 4D, the aggregate/monomer (red/green fluorescence intensity) ratio represented mitochondrial membrane potential depolarization. Quercetin attenuated mitochondrial membrane potential depolarization and restored the decreased ΔΨm in Ang II-induced H9c2 cells in a dose dependent manner (Figure 4E). Moreover, SIRT3 expression was decreased by Ang II, whereas quercetin significantly restored SIRT3 levels, especially in the high-dose group (Figures 4F,G). Therefore, 2 μM quercetin was chosen as the optimal dose for subsequent experiments. These data confirmed that quercetin inhibited the cardiac hypertrophic response and protected mitochondrial function *in vitro*, likely *via* SIRT3 activation.

### Quercetin Protects Mitochondrial Function *via* SIRT3/PARP-1 Pathway

**FIGURE 5:**
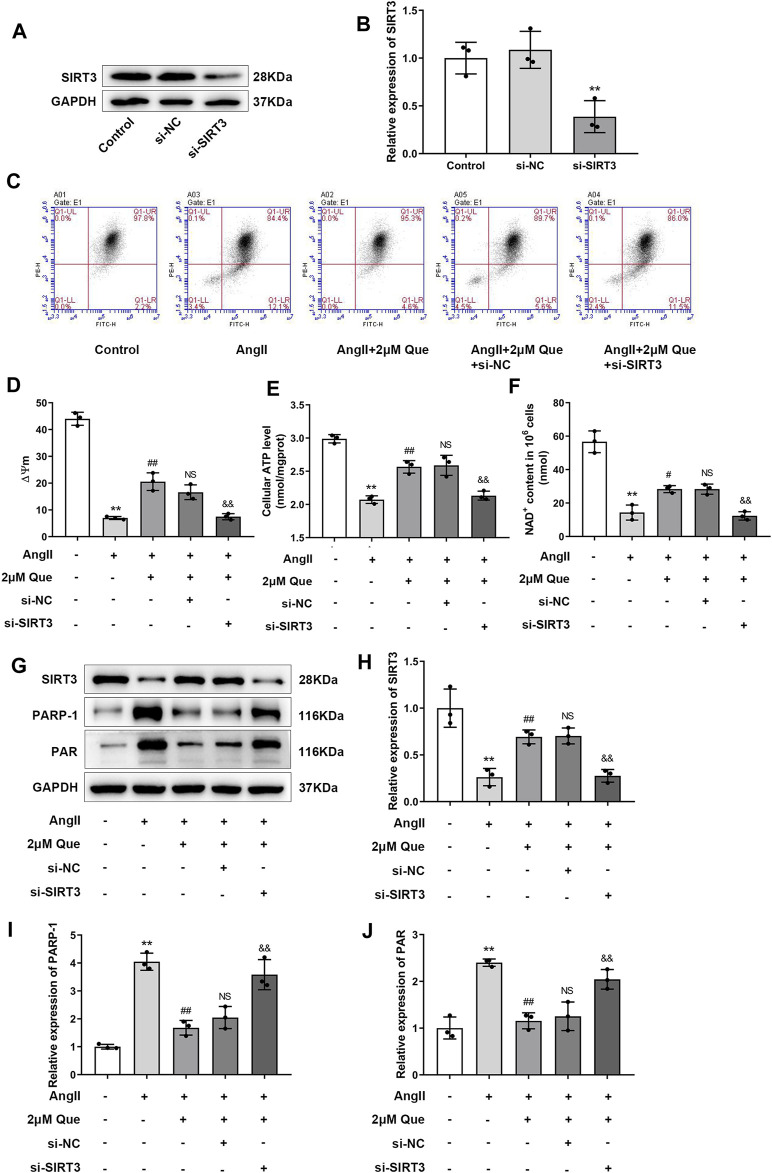
Quercetin protects mitochondrial function *via* SIRT3/PARP-1 pathway. **(A,B)** Representative blots of siRNA-SIRT3-mediated transfection efficiency and densitometric quantification after normalization to GAPDH levels (n = 3). **(C)** ΔΨm measurement with the JC-1 probe. **(D)** Quantification of ΔΨm by the aggregate/monomer ratios (n = 3). **(E)** Quantification of cellular ATP levels with the intracellular ATP Assay Kit (n = 3). **(F)** Introcellular levels of nicotinamide adenine dinucleotide (NAD^+^) determined by NAD^+^/NADH assay kit (n = 3). **(G–J)** Representative blots of SIRT3, PARP-1 and PAR, and densitometric quantification after normalization to GAPDH levels (n = 3). Data are mean ± SD. ^*^ *p* < 0.05, ^**^ *p* < 0.01 *vs* control group; ^#^ *p* < 0.05, ^##^ *p* < 0.01 *vs* Ang II group; ^NS^ *P* > 0.05 *vs* Ang II + 2 μm Que group; ^&^ *p* < 0.05, ^&&^ *p* < 0.01 *vs* Ang II + 2 μm Que group.

Due to the vital roles of SIRT3 in cardiac mitochondrial dysfunction and oxidative stress (Wei et al., 2017), we focused on SIRT3-mediated pathway in quercetin pharmacology using siRNA-SIRT3 to knock down SIRT3. In this study, western blot showed that SIRT3 was effectively knocked down after siRNA transfection (Figures 5A,B). As shown in Figures 5C,D, Ang II induced mitochondrial membrane depolarization and altered the mitochondrial membrane potential in H9c2 cells, which was markedly reversed by quercetin treatment. However, these effects were partly abolished in siRNA-SIRT3 cells but not in siRNA-NC cells. Similar effects were found in the ATP assay (Figure 5E). Since SIRT3 is an NAD^+^-dependent enzyme, its activity is dependent on NAD^+^ level, thus we examined the NAD^+^ level in H9c2 cells. As shown in Figure 5F, quercetin alleviated Ang II-induced NAD^+^ level decrease, which was markedly reduced after SIRT3 knockdown. These results suggested the involvement of SIRT3 activation in quercetin pharmacology in mitochondrial protection.

PARP-1 is an important effector suppressed by SIRT3 overexpression in hypertrophic cardiomyocytes (Feng et al., 2020), its activity is represented by PAR expression. In the present study, quercetin dramatically inhibited PARP-1 and PAR upregulation in Ang II-induced H9c2 cells, whereas this effect was reduced after SIRT3 knockdown (Figures 5G–J). Taken together, these findings demonstrated that quercetin played a protective role in mitochondrial function by regulating SIRT3/PARP-1 signaling pathway.

## Discussion

In the present study, quercetin demonstrated a beneficial effect in attenuating cardiac hypertrophy in SHRs and Ang II-induced hypertrophic response in H9c2 cells, which was concomitant with improved mitochondrial function as well as increased SIRT3 expression both *in vivo* and *in vitro*. The pharmacology of quercetin was studied *via* SIRT3-knockdown by siRNA. The protective effect of quercetin against mitochondrial dysfunction was overtly blocked after SIRT3 knockdown, which was accompanied by PAR upregulation, suggesting that activation of SIRT3/PARP-1 signaling pathway might be the target of quercetin in improving mitochondrial function and attenuating cardiac hypertrophy.

Mitochondria play central roles in the regulation of cellular metabolism responsible for ATP production, ROS generation and detoxification, which are considered to be vital for the maintenance of cellular homeostasis in cardiomyocytes with high-energy demand (Jusic and Devaux, 2020). Accumulating evidence reveals a close relationship between mitochondrial dysfunction and cardiac hypertrophy. During the development of cardiac hypertrophy, the activities of ATP synthase and mitochondrial oxidative phosphorylation complex are decreased, leading to impaired ATP production (Pham et al., 2014). Additionally, in Dahl salt-sensitive rats, hypertensive cardiac hypertrophy and fibrosis are suppressed by mdivi1, an inhibitor of Drp1, *via* reduction of excessive ROS production, which is also associated with improved mitochondrial dynamics (Hasan et al., 2018). Moreover, a recent study demonstrated that diabetic cardiomyopathy in mice shows a reduced IP3-stimulated Ca^2+^ transfer to mitochondria, associated with decreased mitochondrial bioenergetics, indicating disruption of reticulum-mitochondria Ca^2+^ transfer leads to mitochondrial dysfunction in diabetic cardiomyopathy (Dia et al., 2020). Consistent with previous studies, impaired mitochondrial function was found in SHRs and Ang II-induced H9c2 cells in this study.

Quercetin, one of the most distributed bioflavonoids, is widely present in a variety of vegetables and fruits. A growing number of studies have reported beneficial effects for quercetin in cardiovascular disease, cancer, metabolic syndrome, Alzheimer’s disease, and nervous system disorders (Ebrahimpour et al., 2020; Hosseini et al., 2021). In the present study, quercetin decreased blood pressure and cardiac hypertrophy, including cardiomyocyte hypertrophy and cardiac fibrosis as revealed by changed left ventricular structure and enhanced collagen I and α-SMA, *in vivo*. Interestingly, EF and FS in SHRs still increased instead of decreasing, indicating that the heart pathology was still at the hypertrophic stage but not reflecting heart failure. It is possible that quercetin might restore the excessive systolic function of the heart by preventing pressure overload (Chen et al., 2018), but the underlying mechanism needs further investigation. Ang II, the key component of renin-angiotensin system (RAS), plays a vital role in mediating hypertension (Hall, 1991) and cardiac hypertrophy (Sadoshima et al., 1993). During RAS activation, Ang II binds to its specific receptors such as Ang II type-1 receptor (AT1R), which stimulates aldosterone synthesis and release, involving in the pathogenesis of hypertension and cardiac remodeling (Borghi and Rossi, 2015). Thus, Ang II is widely used to establish the cardiac hypertrophic model. ANF and β-MHC, common biomarkers of cardiac hypertrophy, have been used to investigate hypertrophic effects in clinical studies and animal experiments (Liu et al., 2018; Fulgencio-Covián et al., 2020). The results of the *in vitro* experiment showed that Ang II induction led to the increase of ANF and β-MHC at the mRNA level, while quercetin decreased Ang II-induced ANF and β-MHC upregulation. We speculates that RAS inhibition maybe involved in the anti-hypertrophic effect of quercetin. In addition, it is well known that estrogen is a vital regulator in the pathology of hypertension. A recent evidence has revealed that quercetin could bind to type-2 estrogen receptor at a low dose (10 mg/kg/d) intake (Shahzad et al., 2014). Another research confirms that quercetin exerts cardioprotective effects against estrogen receptor α (ERα)-deficiency-induced cardiac dysfunction (Wang et al., 2021). These evidences indicate a close relationship between quercetin and estrogen. In view of this, further study is needed to investigate the involvement of estrogen in the anti-hypertensive effect of quercetin using female rats.

Quercetin has been proven to protect mice cardiac mitochondria against oxidative stress, since quercetin restores mitochondrial GSH levels and elevates Mn-SOD activity (Ballmann et al., 2015). Moreover, quercetin interacts directly with the mitochondrial membrane and the components of the mitochondrial electron transport chain, affecting ATP production (Qiu et al., 2018; Ryu et al., 2019). Since mitochondrial superoxide is the predominant ROS in mitochondria, and excessive mitochdrial ROS production leads to an overwhelmed antioxidant system and oxidative stress (Munro and Treberg, 2017). Thus we evaluated the anti-oxidant effcts of quercetin by measuring mitochondrial superoxide. In this study, quercetin protected cardiac mitochondria against morphological impairment as well as increased mitochondrial superoxide and oxidative stress in SHRs. In addition, membrane potential disruption and ATP collapse caused by Ang II in cultured H9c2 cells were also rescued by quercetin, indicating that quercetin improves mitochondrial function by alleviating oxidative stress and restoring ATP production.

SIRT3, one of the most prominent deacetylases, is mainly found in the mitochondrial matrix, playing a vital role in the mitochondrial metabolism and homeostasis by modulating the acetylation levels of its substrates, thus protecting mitochondria from functional disorders. Accumulating evidence reveals a close link between SIRT3 and human diseases, including age-related diseases, cancer, cardiovascular diseases and metabolic diseases (Zhang et al., 2020). Among them, the role of SIRT3 in cardiac hypertrophy has attracted considerable attention. A previous study showed that SIRT3 deficiency impairs mitochondrial function and accelerates hypertensive cardiac remodeling through mitochondrial oxidative stress (Wei et al., 2017). Another study reported that SIRT3 attenuates diabetic cardiomyopathy by regulating p53 acetylation and TIGAR expression (Li et al., 2021). PARP-1 is involved in the pathological mechanism of cardiac hypertrophy and heart failure. It was reported that PARP-1 expression is significantly elevated in the onset and progression of cardiac hypertrophy (Pillai et al., 2005). In addition, the PARP-1 inhibitor AG-690/11026014 (6,014) improves Ang II-induced cardiac remodeling and function in mice. Moreover, another PARP-1 inhibitor, L-2286, can not only prevent the development of left ventricular hypertrophy in SHRs, but also modulate mitochondrial dynamics and biogenesis, indicating that PARP-1 may be a therapeutic target in hypertensive cardiac hypertrophy.

In the current study, both SIRT3 expression and NAD^+^ content were decreased in Ang II-induced H9c2 cells, indicating that Ang II stimulated hypertrophic response partly through inhibiting SIRT3 activity. But these changes were reversed after quercetin treatment. To further investigate the role of SIRT3 in quercetin pharmacology, SIRT3 was knocked down using small interfering RNA (siRNA). Normal and siRNA H9c2 cells were treated with Ang II with or without quercetin. We found that after SIRT3 knockdown, the downregulation effect of quercetin on PARP-1 activity was partly abolished. Meanwhile, mitochondrial protection by quercetin (oxidative stress and ATP production) was blocked after SIRT3 knockdown. These results suggested that quercetin protects mitochondrial function by modulating SIRT3/PARP-1 signaling pathway, which is involved in the prevention of cardiac hypertrophy.

In summary, the present study provides evidence that quercetin ameliorates cardiac hypertrophy by protecting mitochondrial structure and function in SHRs and Ang II-induced H9c2 cells, involving the SIRT3/PARP-1 pathway. These findings provide a potentially mechanic insight into quercetin-mediated attenuation of cardiac hypertrophy.

## Limitation

Although we evaluated mitochondrial function using ΔΨm and ATP production *in vitro*, the biological process of mitochondrial function is complicated, and there are several other assays for assessing mitochondrial function. Thus, the pharmacological mechanisms of quercetin’s effects on mitochondrial function and cardiac hypertrophy deserve further investigation. Futhermore, we used Ang II as a stimuli to trigger the hypertrophic response *in vitro*, but we didn’t detect the expression of RAS in Ang II-induced H9c2 cells. This issue should be further studied in our future work.

## Data Availability

The raw data supporting the conclusions of this article will be made available by the authors, without undue reservation.
