# Supplementary material for: Quercetin Attenuates Cardiac Hypertrophy by Inhibiting Mitochondrial Dysfunction Through SIRT3/PARP-1 Pathway
Source: Front Pharmacol. 2021 Oct 28;12:739615. doi: 10.3389/fphar.2021.739615 (PMC8581039; doi:10.3389/fphar.2021.739615)
Supplement: Supplementary file 1 [file Table1.DOCX]

Supplementary Table 1. Primer sequences for q-PCR.

| **Primer** | **Sequences** |
| --- | --- |
| ANF | 5'-CTGGGACCCCTCCGATAGAT-3' (Forward) |
|  | 5'-GTCAATCCTACCCCCGAAGC-3' (Reverse) |
| β-MHC | 5'-GTCCCGAGGTGTACTTTCCA-3' (Forward) |
|  | 5'-GCTTCATCCACGGCCAATTC-3' (Reverse) |
| GAPDH | 5'-GCGAGATCCCGCTAACATCA-3' (Forward) |
|  | 5'-CTCGTGGTTCACACCCATCA-3' (Reverse) |
