# Supplementary material for: Quercetin Attenuates Cardiac Hypertrophy by Inhibiting Mitochondrial Dysfunction Through SIRT3/PARP-1 Pathway
Source: Front Pharmacol. 2021 Oct 28;12:739615. doi: 10.3389/fphar.2021.739615 (PMC8581039; doi:10.3389/fphar.2021.739615)

Supplemental figure S1： Original blots of cropped images with three samples shown in Figure 2I and 2K (captured with film imaging)

Lane 1: control group (WKY)

Lane 2: SHR group

Lane 3: Quercetin group

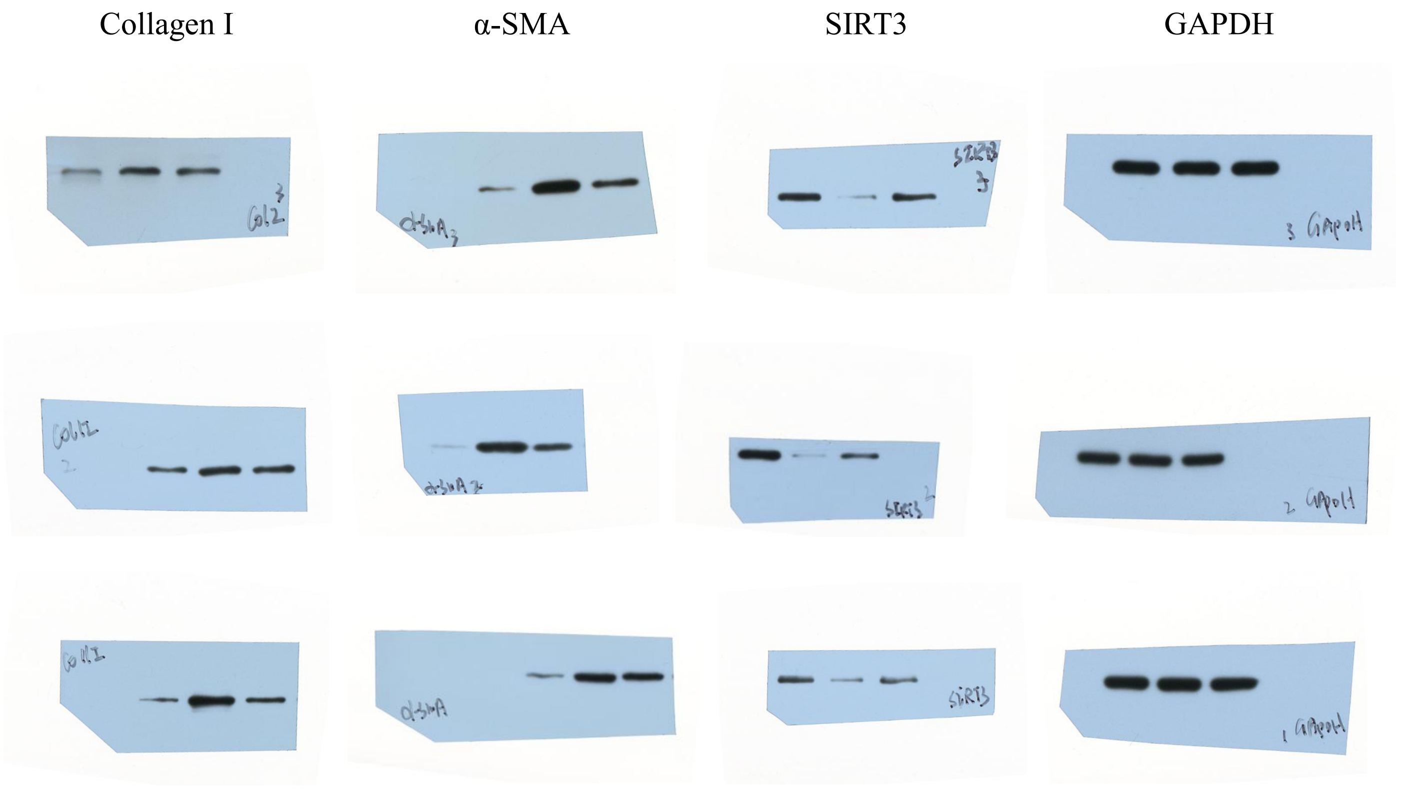

Supplemental figure S2： Original blots of cropped images with three replicates shown in Figure 4F (captured with gel imaging system)

Lane 1: marker

Lane 2: control group

Lane 3: Ang II group

Lane 4: Ang II + 0.5 μM quercetin group

Lane 5: Ang II + 1 μM quercetin group

Lane 6: Ang II + 2 μM quercetin group

SIRT3 GAPDH

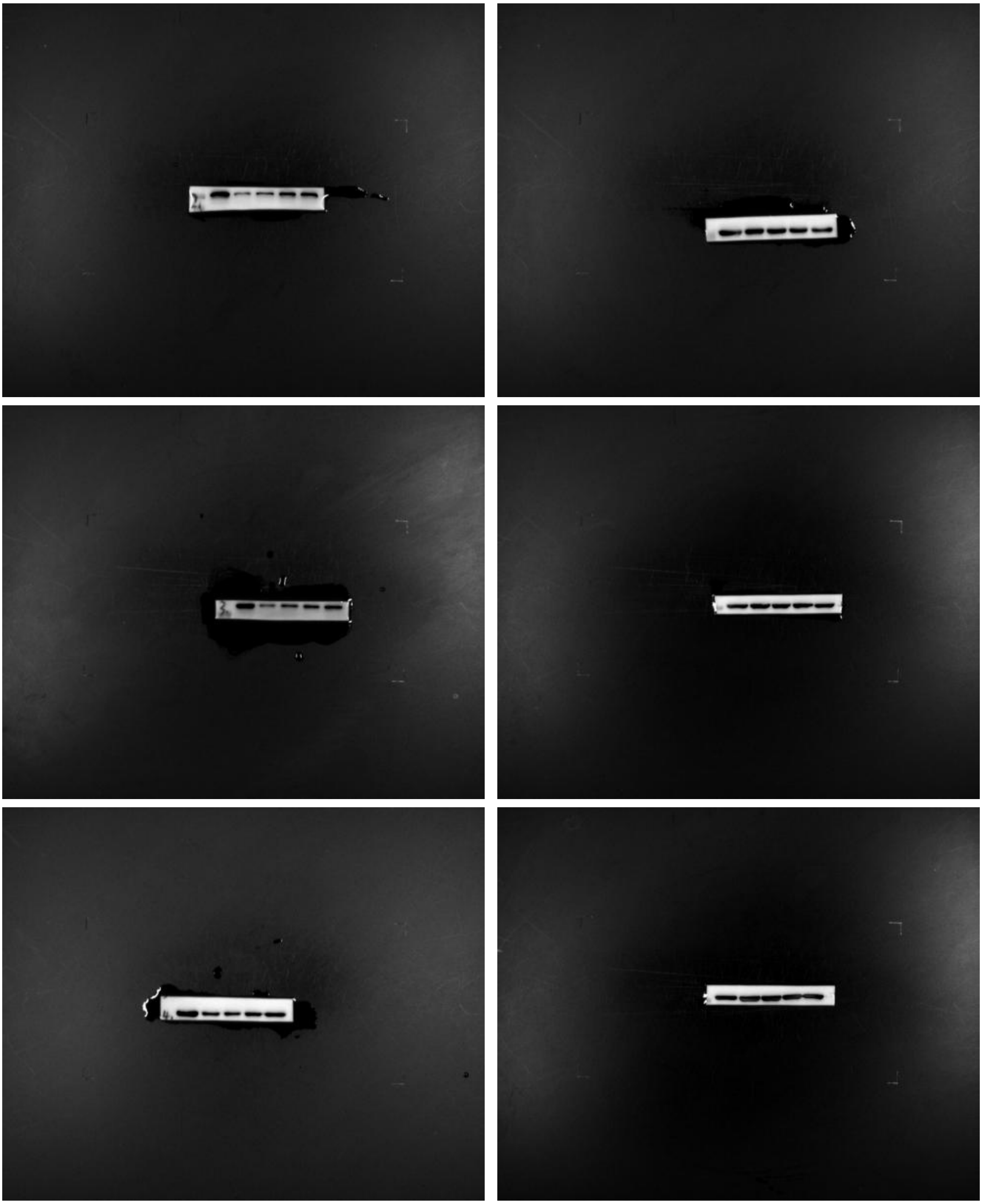

Supplemental figure S3： Original blots of cropped images with three replicates shown in Figure 5A (captured with gel imaging system)

Lane 1: marker

Lane 2: control group

Lane 3: siRNA-NC group

Lane 4: siRNA-SIRT3 group

SIRT3 GAPDH

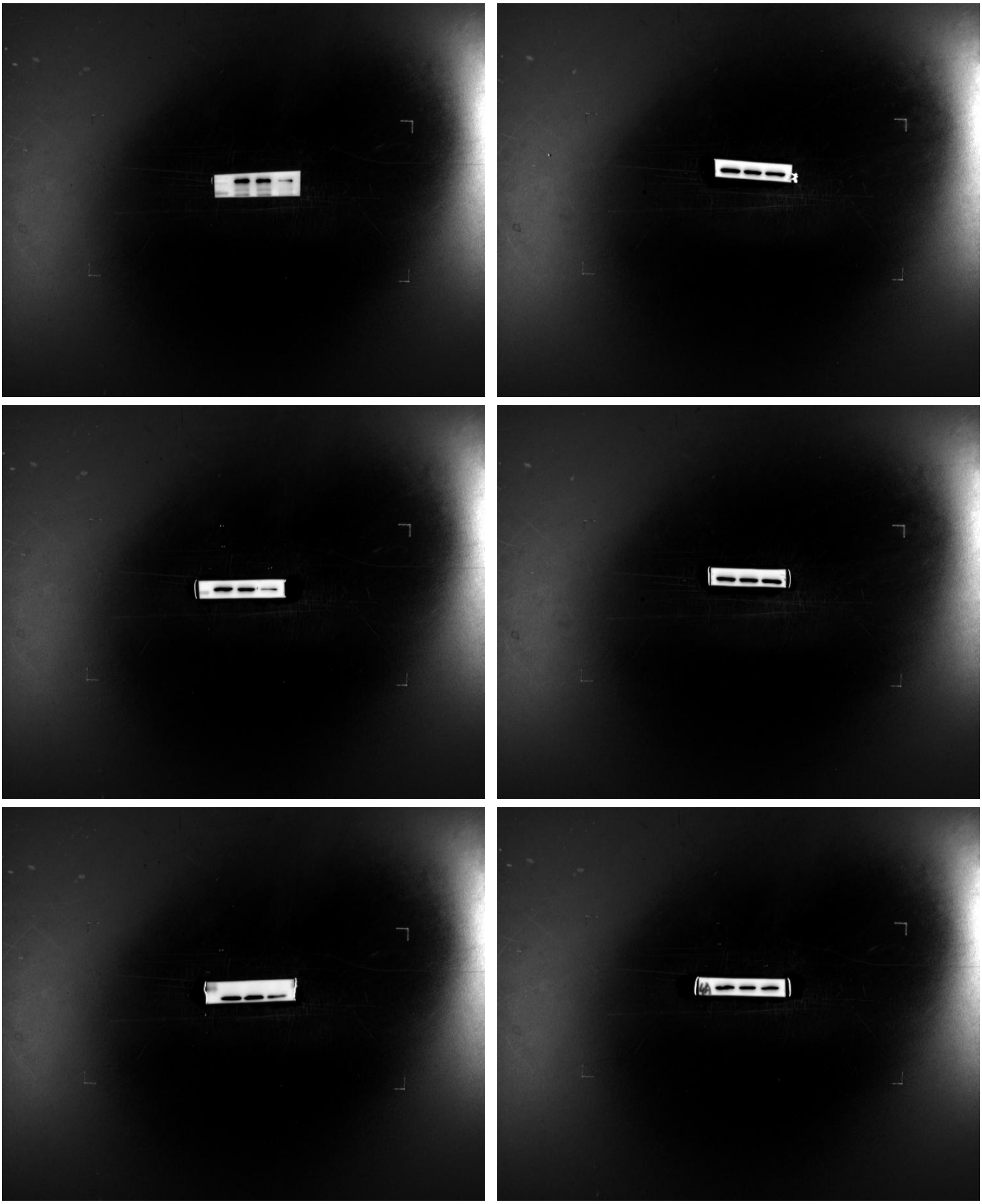

Supplemental figure S4： Original blots of cropped images with three replicates shown in Figure 5G (captured with gel imaging system)

Lane 1: marker

Lane 2: control group

Lane 3: Ang II group

Lane 4: Ang II + 2 μM quercetin group

Lane 5: Ang II + 2 μM quercetin + siRNA-NC group

Lane 6: Ang II + 2 μM quercetin + siRNA-SIRT3 group

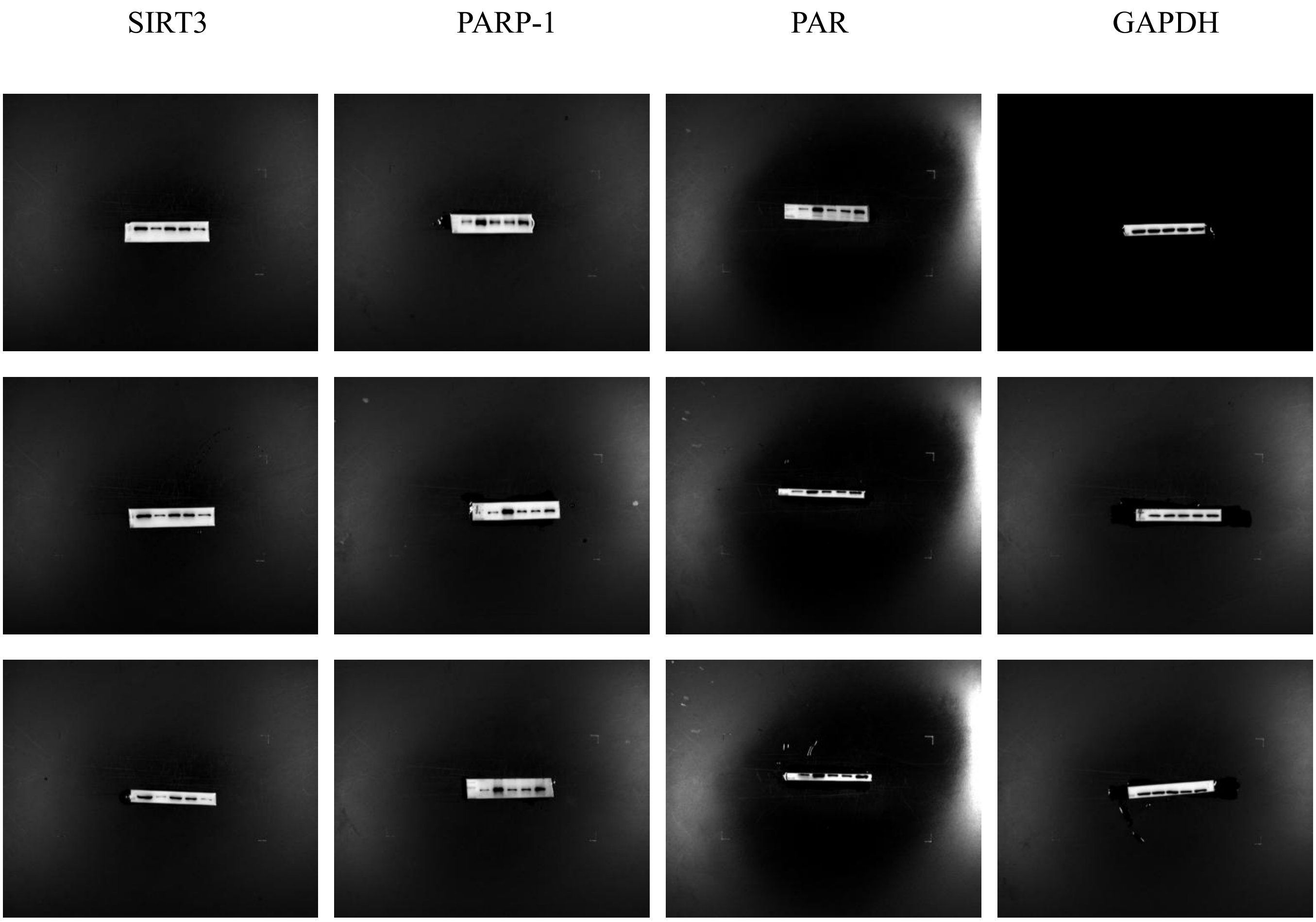

Supplement: Supplementary file 2 [file DataSheet1.PDF]
